# Extracorporeal Membrane Oxygenation for COVID-19: A Systematic Review

**DOI:** 10.7759/cureus.27522

**Published:** 2022-07-31

**Authors:** Rawah Shafiq Aljishi, Ali Hussin Alkuaibi, Fadel Abbas Al Zayer, Ali Hassan Al Matouq

**Affiliations:** 1 Critical Care Medicine, King Salman Hospital, Riyadh, SAU; 2 Critical Care Medicine, King Fahad Medical City, Riyadh, SAU; 3 Critical Care Medicine, King Faisal Specialized Hospital and Research Center, Riyadh, SAU

**Keywords:** complications, mortality, ards, covid-19, extracorporeal membrane oxygenation, ecmo

## Abstract

COVID-19 may result in acute respiratory distress (ARDS) in patients with the severe form of the disease. Extracorporeal membrane oxygenation (ECMO) can support respiratory gas interchange in patients failing conventional methods, but its effectiveness in COVID patients is still debatable.

The aim of this study is to find the survival outcomes of patients with and without COVID-19 ARDS who were supported with extracorporeal membrane oxygenation (ECMO). PubMed, Medline, and Google Scholar databases were searched from 2020 to 2022. Studies comparing the outcomes of ECMO in COVID and non-COVID ARDS were included. The outcomes that were measured were mortality or survival, survival to discharge, ECMO duration, and complications. This systematic review encompassed 12 retrospective observational studies and one quasi-controlled trial, including a total of 12 studies that recruited 1,133 patients (495 COVID-19 and 638 non-COVID ARDS patients) and were published between 2020 and 2022.

The overall mortality rate of ECMO-supported COVID-19 patients was 41% and ranged between 14.7% and 67%. On the other hand, non-COVID ARDS patients' mortality rate ranges from 14.3% to 50%. In comparison, COVID-19 patients had a prolonged duration of ECMO therapy as well as increased bleeding and thrombotic complications. Our findings suggest that ECMO remains a viable option for the management of COVID-19-associated acute respiratory distress syndrome for selected patients. The observed mortality rate was 41%. Meta-analyses are warranted to obtain more conclusive results and assess the risk.

## Introduction and background

The 2019 outbreak of severe acute respiratory syndrome coronavirus 2 (SARS-CoV-2) rapidly evolved into a pandemic. COVID-19 (coronavirus disease 2019) can cause acute respiratory failure, necessitating admission to an intensive care unit (ICU) and assisted ventilation. Despite lung-protective mechanical ventilation, its most serious variants can rapidly progress into acute respiratory distress syndrome (ARDS) with multi-organ failure and death [1, 2].

The Extracorporeal Life Support Organization (ELSO) suggests that COVID-19 patients suffering from acute cardiopulmonary impairment should be put on extracorporeal membrane oxygenation (ECMO). In addition, the World Health Organization suggested that the specialist units with appropriate ECMO volume maintain proficiency to consider ECMO assistance in COVID-19-related ARDS with refractory hypoxemia if lung-protective mechanical ventilation is ineffective [3].

Venovenous (V-V) ECMO improves 90% of COVID-19 patients with ARDS. Venovenous ECMO is an invasive technique that oxygenates the blood and removes CO_2_ while the failing lung is rested and is given time to recover [4]. Initial research revealed that COVID-19 patients receiving ECMO had a higher mortality rate [5], even though some cohort investigations reported that ECMO outcomes did not show a significant difference between COVID-19 and other types of ARDS [6, 7]. It encourages health care professionals to provide ECMO when deemed appropriate. In this systematic review, we investigated mortality or survival outcomes along with ECMO duration and complications of ECMO-supported patients among COVID-19 and non-COVID-19 ARDS patients.

## Review

Methods

Definition of Outcomes and Inclusion Criteria

We aimed to investigate the clinical outcome of ARDS patients with COVID-19 who were placed on ECMO. The clinical outcomes include mortality, survival, ECMO duration, and complications. We included the original investigations that recruited ARDS COVID-19 patients who underwent ECMO support. The present review includes studies comparing non-COVID ARDS groups with COVID-19 ARDS groups. Studies without comparison groups, case reports, or case series with limited sample sizes, and those without descriptive statistics were excluded from this review. Other exclusion criteria included duplicate data, data not related to COVID-19, data not containing the outcomes of measures, non-original investigations or incomplete studies, abstract-only articles, protocols, theses, and articles that weren’t published in English or with no available information in English.

Search Strategy

Relevant literature was searched in multiple databases, including PubMed, Medline, and Google Scholar. The following specific keywords were used alone or in combination for the search: ARDS, ECMO, COVID-19, and non-COVID ARDS. Boolean operators (AND, OR, NOT) were also used to increase the sensitivity of the search. Our search strategy was limited to the title and abstract of the search results to utilize all the relevant studies only. All of these results were exported to an Endnote library to identify and execute all duplicates between the different search databases. Furthermore, we manually searched all similar article sections in PubMed and included studies and relevant reviews for possible detection of any missed studies by the main electronic search strategy. The current systematic review follows the Preferred Reporting Items for Systematic Reviews and Meta-Analysis (PRISMA) standards.

Screening and Extraction

We performed a double screening strategy-one for screening titles and abstracts and the other for screening full texts to maintain high quality in this important process. After ensuring that all relevant articles were included, an extraction sheet was constructed in an organized way relevant to our aimed outcomes. The sheet was composed of the baseline characteristics and the sought outcomes and complications.

Quality Assessment

We utilized the modified Newcastle-Ottawa scale (NOS) to assess the bias in non-randomized observational studies recommended by Cochrane collaborations [8]. This tool has three domains: Study group recruitment, group comparability, and exposure and outcome measurement. The conversion thresholds from the Newcastle-Ottawa scale to Agency for Healthcare Research and Quality standards are as follows: three or four markings in the selection domain, one or two markings in the comparison domain, and two or three markings in the outcome/exposure domain indicate good quality. Two markings in the selection domain AND one or two markings in the comparability domain and two or three markings in the outcome/exposure domain equals fair quality, while zero or one mark in the selection domain OR zero marks in the comparability domain OR zero or one mark in the outcome/exposure domain equals poor quality.

Results

Search Results

**Figure 1:**
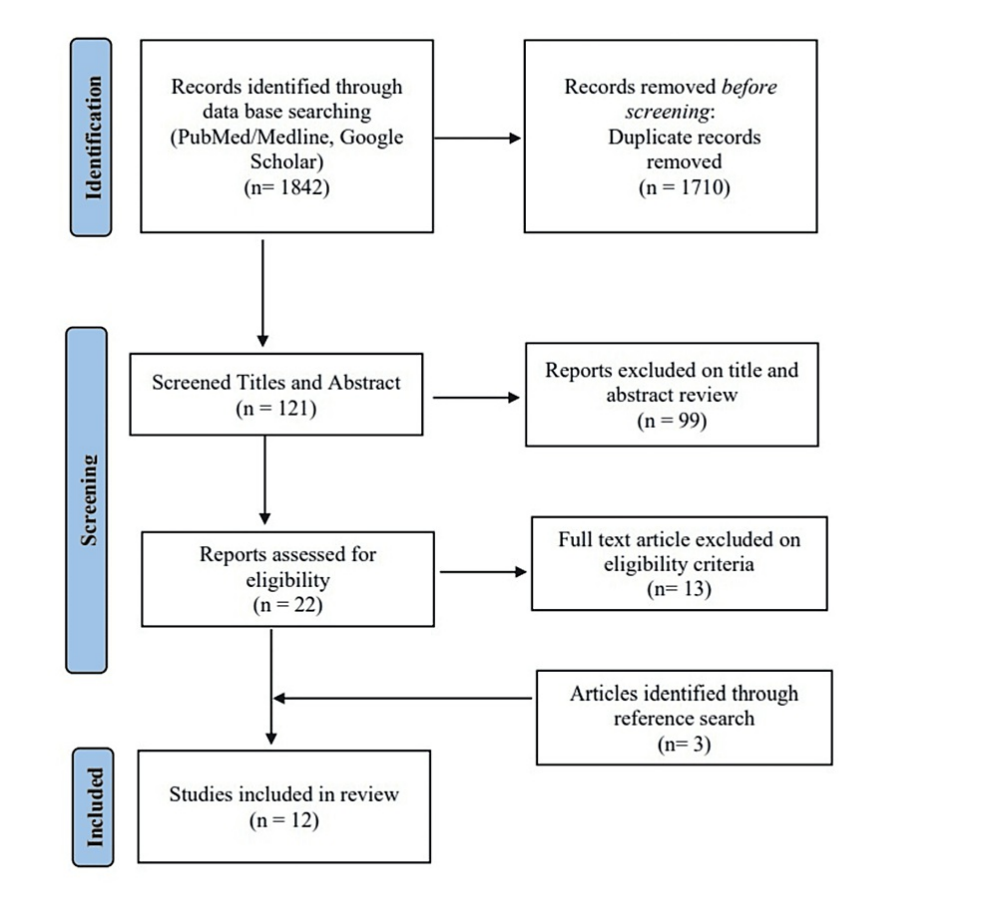
PRISMA flow diagram of the included studies

By conducting the aforementioned search strategies, we managed to find a total of 1842 citations which were then shortened to 132 after the removal of duplicates. Following title and abstract screening, only 22 citations were eligible for the next steps. Full-text screening showed that only nine articles matched our inclusion and exclusion criteria. An examination of references turned up three additional studies that met our inclusion criteria and were added to the systematic review. There was a total of 12 observational research investigations that matched the inclusion criteria, totaling 1133 patients (Figure 1).

Results of the Quality Assessment

Among the included studies, 12 studies had clear aims and outcomes, and all the studies were retrospective in nature. Because neither of the included research used prospective randomized analyses, most of the studies were rated as fair quality, while three studies were rated as good quality on the Newcastle-Ottawa scale (Table 1).

**Table 1:** Summary of the results of bias assessment of the included studies using the modified Newcastle-Ottawa scale (NOS) for non-randomized observation studies * low risk of bias, ** medium risk of bias, *** high risk of bias

No	Author	Selection of the study group	Comparability	Ascertainment of the exposure and outcome	Total Score	Study Quality
1	Gurnani PK [9]	***		**	5	fair
2	Fanelli V [10]	***	*	**	6	good
3	Cousin N [11]	***		**	5	fair
4	Raasveld SJ [12]	***	*	**	6	good
5	Raff LA [13]	***	*	**	6	good
6	Kurihara C [14]	***		**	5	fair
7	Bemtgen X [15]	***		**	5	fair
8	Gjurašin B [16]	***		**	5	fair
9	Luyt CE [17]	***		**	5	fair
10	Charlton M [18]	***		**	5	fair
11	Jäckel M [19]	***		**	5	fair
12	Russ M [20]	***		**	5	fair

Characteristics of the Included Studies

We looked at 12 studies that recruited 1,133 patients (495 COVID-19 and 638 non-COVID ARDS patients) and were published between 2020 and 2022 and accounted for 60% of the male population. We included a total of six clinical trials that were conducted in Germany and the United States. In addition, we included multicenter investigations. Lastly, France, the United Kingdom, and Croatia each had one study accordingly (Table 2).

**Table 2:** Baseline characteristics of the included studies in this review NR: not reported, ^a ^COVID-19; ^b ^non-COVID, VV ECMO: venovenous ECMO

No	Author	Year	Country	Study Design	Study Type	Type of ECMO	Sample size		Age	Gender
							COVID	Non-COVID		
1	Gurnani PK [9]	2022	USA	Observational	Retrospective	VV ECMO	34	28	45.5 (39.5–51.3)^a,^ 48.5 (36.0–54.8)^b^	23/11^a^, 13/15^b^
2	Fanelli V [10]	2022	Multicentre	Observational	Retrospective	VV ECMO	146	162	53 (48–59)^a, ^47 (37–58)^b^	124/22^a^,100/62^b^
3	Cousin N [11]	2021	France	Observational	Retrospective	VV ECMO	30	22	57 (47–62)^a^, 55 (48–60)^b^	24/6^a^,14/8^b^
4	Raasveld SJ [12]	2021	Multicentre	Observational	Retrospective	VV ECMO	71	48	52 (47-57)^a^, 55 (40-61)^b^	57/14^a^, 24/24^b^
5	Raff LA [13]	2021	USA	Observational	Retrospective	VV ECMO	32	28	47.8 (10.3)^a^, 41.2 (12.8)^b^	25/7^a^,16/12^b^
6	Kurihara C [14]	2021	USA	Observational	Retrospective	VV ECMO	26	112	47.6 ± 10.9^a^, 47.8 ± 15.3^b^	19/7^a^,63/49^b^
7	Bemtgen X [15]	2021	Germany	Observational	Retrospective	VV ECMO	11	55	58.4 (46.9–66.2)	7/4^a^,33/22^b^
8	Gjurašin B [16]	2020	Croatia	Observational	Retrospective	VV ECMO	30	42	70 (56–75)^a^, 55 (45–63)^b^	22/8^a^, 20/22^b^
9	Luyt CE [17]	2020	France	Observational	Retrospective	VV ECMO	50	45	48 (42–56)^a^, 58 (48–64)^b^	36/14^a^,28/17^b^
10	Charlton M [18]	2020	UK	Observational	Retrospective	not mentioned	34	26	46.3 (7.5)^a^, 43.1 (8.7)^b^	27/7^a^,18/8^b^
11	Jäckel M [19]	2020	Germany	Observational	Retrospective	VV ECMO	15	47	60.8 (54.1-67.0)^a^, 52.7 (41.9-60.7)b	11/4^a^,28/19^b^
12	Russ M [20]	2022	Germany	Observational	Retrospective	VV ECMO	16	23	At least 48-year-old	NR

Outcomes of ECMO in COVID-19 and Non-COVID ARDS Patients

A total of 11 included studies reported [9-19] mortality rates among COVID-19 and non-COVID-19 ARDS patients who received ECMO support. The mortality rate ranged from 14.7% to 65.62% in COVID-19 patients, while in non-COVID ARDS patients it ranged from 14.3% to 50%. Even though the number of cases with mortality was higher in COVID-19 patients, the majority of the included studies found no statistical difference between the COVID and non-COVID ARDS patients' mortality who were placed on ECMO support. This indicates that COVID ARDS patients had the same benefit as other ARDS patients with ECMO. However, COVID-19 patients required longer ECMO support compared to non-COVID-19 (Table 3). Survival rate was compared in five included studies [9, 13, 15, 19, 20]. Two studies reported 28 days and 30 days of survival after ECMO support [15, 19], two studies reported survival of patients to hospital discharge [9, 13], and one study reported ICU survival rate in ECMO supported patients [20]. Moreover, 28 days and 30 days of survival were similar in both groups and did not show any significant difference between the groups. There was similar survival to hospital discharge between the COVID and non-COVID awake ECMO patients [9]. One study reported crude in-hospital mortality that was significantly higher in the COVID-19 versus the non-COVID cohort [13]. Complications were evaluated in six of the included studies [10-15]. The COVID-19 group had more hemorrhagic and thrombotic complications (Table 3).

**Table 3:** Summary of the outcomes of the included studies in this review ICU: intensive care unit, NR: not reported, ^a^ COVID-19; ^b^ non-COVID

No	Author	Mortality n (%)		Survival to discharge/ICU n (%)		ECMO days		Complications
		COVID-19	Non-COVID	COVID-19	Non-COVID	COVID-19	Non-COVID	
1	Gurnani PK [9]	5 (14.7%)	4 (14.3%)	29 (85.3%)	24 (85.7%)	49 (25–87)	22 (14–38)	
2	Fanelli V [10]	67 (46%)	43 (27%)	NR	NR	22 (11–38)	13 (9–22)	Hemorrhagic complications: 47%^a^ ,31%^b^
3	Cousin N [11]	13 (43.3%)	11 (50.0%)	14 (47%)	12 (55%)			Bleeding: 22 (73.3%)^a^, 14 (63.6%)^b^, thrombosis: 10 (33.3%)^a^, 3 (13.6%)^b^
4	Raasveld SJ [12]	26 (37%)	27%	NR	NR	13 (7-20)	9 (5-17)	Hemorrhagic complication 38/71^a^ 22/46^b^, arterial thrombosis: 3/71^a^, venous thrombosis: 8/71^a^,4/48^b^, mechanic thrombosis: 10/71^a^, 7/48^b^, infection: 40/56^a^, 26/48^b^
5	Raff LA [13]	21 (65.62%)	11 (39.28%)	11 (34.37%)	17 (61%)	12.4 (5.7)	7.7 (5.1)	Bleeding, 22 (68.8%)^a^,19 (67.9%)^b^
6	Kurihara C [14]	12 (46%)		NR	NR	NR	NR	Bleeding: 12 (46.1%)^a^, 61 (54.4%)^b^, thrombotic complication:12 (46.1%)^a^ 27 (24.1%)^b^
7	Bemtgen X [15]	3 (27.27%)	23 (41.82%)	8 (72.7%)	32 (58.2%)	17.94 (7.8–23.75)	7.49 (4.15–16.34)	Pump head thrombosis: 9/11^a^: 16/55^b^
8	Gjurašin B [16]	19 (63%)	23 (55%)	NR	NR	17 (13–26)	13 (7–25.5)	NR
9	Luyt CE [17]	17 (34%)	18 (40%)	2	7	21 (10–34)	18 (8–31)	NR
10	Charlton M [18]	16 (47%)	8 (31%)	NR	NR	13.2 (5.6)	12.3 (8.0)	NR
11	Jäckel M [19]	7 (46.7%)	18 (38.3%)	13.3%	44.7%	11.3 (7.8-23.8)	8.9 (4.8-15.1)	NR
12	Russ M [20]	NR	NR	62%	70%	43 (18–58)	16 (19–39)	NR

Discussion

Mortality and Survival

The use of ECMO in adult patients with and without COVID-19 ARDS was explored in this systematic review. Early reports from China on the use of V-V ECMO in COVID-19 demonstrated mortality as high as 83%, although the studies were modest, limiting the capacity to draw solid conclusions [21, 22]. Information detailing outcomes in patients receiving ECMO is limited to case reports and survival outcomes. The early studies evaluating the efficacy of ECMO and comparing the outcomes with non-COVID ARDS were small and inconclusive. Therefore, in this systematic review, the primary investigated outcome was mortality or survival of ECMO-supported patients among COVID and non-COVID ARDS patients. According to the CDC guidelines, ECMO should be explored as a viable therapy as part of the standard management strategy for COVID-19-associated ARDS patients in areas where it is accessible [23]. Using the meta-analysis results, Ramanathan et al. [24] stated that the in-hospital death rate in patients who received ECMO support for COVID-19-related ARDS was 37.1% and that ECMO seems to be an efficient approach in selected COVID-19-related ARDS patients. Bertini et al. [25] found a 37% mortality rate in COVID patients who utilized ECMO in a systematic review published in 2021. In our review, the overall mortality rate of COVID-19 patients was 41% and ranged between 14.7% and 65.62%. Only two of the manuscripts we looked at had a mortality rate of higher than 50%. The lower rate of overall mortality implies that ECMO could be advantageous for patients with COVID-19 with ARDS. Branimir et al. [16] discovered that patients with severe influenza and COVID-19 had increased mortality in both research groups (55% and 63%). According to Piroth et al., the increased rate of mortality among COVID-19 patients was not the result of an influenza season, which was less severe than usual [26]. Therefore, the fact that many individuals had the most severe form of ARDS with frequent and expected adverse outcomes explains the greater mortality in COVID-19. Furthermore, we compared the death or survival outcomes of COVID-19 patients to non-COVID patients with ARDS in ECMO-supported cases in our review. Hospital mortality, the chance of needing invasive mechanical ventilation, and ICU duration of stay are three times higher in COVID-19 patients than in influenza patients, according to large cohort research [26]. The 90-day death rate in the ECMO group was significantly lower, as per a meta-analysis of the CESAR [27] and EOLIA [28] randomized control trials that compared ECMO with conventional care in ARDS patients. However, due to resource limits, ECMO may not be a therapy that can be widely administered, but judicious use of appropriately selected patients could be highly successful [29].

Many centers had high criteria for commencing ECMO in the early phases of the pandemic. The demand for ECMO grew in the months that followed, and intensivists struggled to find the best candidates for ECMO. Early-adopter locations used the prone posture and neuromuscular blockade more frequently during the pandemic [24]. Before May 1, 2020, 37% of ECMO patients died, compared to 52% at early adopter hospitals and 59% at late adopter sites after that date [30]. COVID-19 management has improved as the clinicians gained more experience treating the distinctive characteristics of the virus along with the development of newer COVID-19 therapies. Over time, ECMO-related outcomes may also improve for patients with severe COVID-19. Raff et al. reported increased mortality for COVID-19 patients on ECMO that is higher than that currently reported by ELSO and numerous other single-center studies [13]. They explained that poor patient selection such as age or pre-ECMO duration of mechanical ventilation may be the factors related to worse outcomes. The use of a ventilator for more than seven days before starting V-V ECMO was likewise linked to greater mortality [14]. This should be taken into account when determining whether a COVID-19 patient will benefit from V-V ECMO. This research implies that rather than the varied viral etiologies, the outcome of cases with ARDS caused by viral infection may be determined by patient selection.

According to Russ et al., COVID-19 patients had a better survival rate than non-COVID patients [20]. Gurnani et al. discovered that COVID and non-COVID awake ECMO patients had equal survival to discharge (85% in two groups, p = 1.000) [9]. In line with this, Bemtgen et al. reported no significant difference in COVID- and non-COVID patients' survival rates (72.7% vs. 58.2% p=0.505) [15]. Jäckel et al. found no significant differences in 30-day survival rates (48.6 % in COVID-19 patients, 63.7% in influenza patients; P =.23) [19]. These findings emphasize the importance of an ECMO strategy for achieving a higher discharge survival.

ECMO Duration

All our included studies explained that patients in the COVID-19 group did have a longer duration of ECMO. By day 14, COVID-19 ECMO survivors remained on ECMO, whereas, 20% of the non-COVID-19 ECMO survivors had already been weaned off. COVID-19 patients often require extended time in intensive care units even after effective ECMO weaning. Most patients are kept on ECMO for a while and then require additional care to recover from severe delirium caused by long periods of sedation needed for mechanical ventilation and ECMO support, along with profound weakness caused by the use of neuromuscular blocking agents while on mechanical ventilation [31-33]. Patients that were placed on ECMO and developed COVID-19-related ARDS and had a prolonged course on ECMO experienced long-term respiratory complications. Continuous risk-benefit analysis of ECMO therapy is required.

Complications

Six included studies documented complications during V-V ECMO in COVID‑19 patients. The complication rate was significantly higher in COVID-19 patients. Complications of cannula insertion for ECMO included hemorrhagic problems, thrombocytopenia (heparin-induced or others), neurologic injury (from hypoxemia or thrombosis), and cannula-related vascular complications, which were found to be higher in the COVID-19 group (Table 3). Bemtgen et al. [15] compared the rate of thrombotic circuit problems in COVID-19 V-V ECMO patients to a retrospective group of patients between 2018 and 2019 (9/11 versus 16/55, respectively, p<0.01). However, Raasveld et al. [12] found that their complications such as hemorrhagic arterial thrombosis venous thrombosis, pump head thrombosis, and infection rates did not significantly differ from their non-COVID-19 peers on ECMO. Raff et al. [13] also confirmed that major and minor bleeding complications did not differ between COVID and non-COVID ARDS cases. However, COVID-19 patients supported with V-V ECMO had a higher incidence of bleeding and thrombotic complications explained by Kurihara et al. [14]. As per emerging results, COVID-19 tends to be connected to a high rate of venous and arterial thromboembolic events, with a reported cumulative incidence of 20% [34-36]. After reports of thrombotic issues in COVID-19, the anticoagulation target has been modified from aPTT (activated partial thromboplastin time) 40-50 s to aPTT 50-70 s [37].

The variability in ECMO initiation and treatment among study sites and countries, as well as additional variability during the pandemic, may have added increased heterogeneity. At the time of publishing, the endpoints of cases who were still in the hospital or on ECMO were unknown. Given that the majority of these investigations were single-center retrospective studies with no risk adjustment or propensity score weighting, these factors could have brought various confounders. Another limitation of the outcomes was that some studies were followed up until 90 days after weaning from ECMO while some study endpoint was measured till ICU discharge/hospital discharge. In the end, the studies still show that ECMO has potentially high mortality rates.

## Conclusions

Our findings suggest that ECMO remains a viable option for the management of COVID-19-associated ARDS for selected patients. Regarding discharge rate, ECMO did not have a significant effect on early discharge rates. However, long-term COVID-19 patients had a better survival rate. Nonetheless, ECMO still presents a high-risk step due to its variety of complications. For COVID-19 patients, V-V ECMO still has a high risk of mortality when used in those who developed ARDS but improves survival rates among those who had a longer COVID-19 course.
